# Identification of Eusynstyelamide B as a Potent Cell Cycle Inhibitor Following the Generation and Screening of an Ascidian-Derived Extract Library Using a Real Time Cell Analyzer

**DOI:** 10.3390/md12105222

**Published:** 2014-10-17

**Authors:** Michelle S. Liberio, Martin C. Sadowski, Colleen C. Nelson, Rohan A. Davis

**Affiliations:** 1 Eskitis Institute for Drug Discovery, Griffith University, Nathan, Qld 4111, Australia; E-Mail: michelle.liberio@griffithuni.edu.au; 2 Australian Prostate Cancer Research Centre—Queensland, Institute of Health and Biomedical Innovation, Queensland University of Technology, Princess Alexandra Hospital, Translational Research Institute, Brisbane, Qld 4102, Australia; E-Mails: martin.sadowski@qut.edu.au (M.C.S.); colleen.nelson@qut.edu.au (C.C.N.)

**Keywords:** ascidian, eusynstyelamide B, cytotoxic, MDA-MB-231, RTCA, G2/M arrest

## Abstract

Ascidians are marine invertebrates that have been a source of numerous cytotoxic compounds. Of the first six marine-derived drugs that made anticancer clinical trials, three originated from ascidian specimens. In order to identify new anti-neoplastic compounds, an ascidian extract library (143 samples) was generated and screened in MDA-MB-231 breast cancer cells using a real-time cell analyzer (RTCA). This resulted in 143 time-dependent cell response profiles (TCRP), which are read-outs of changes to the growth rate, morphology, and adhesive characteristics of the cell culture. Twenty-one extracts affected the TCRP of MDA-MB-231 cells and were further investigated regarding toxicity and specificity, as well as their effects on cell morphology and cell cycle. The results of these studies were used to prioritize extracts for bioassay-guided fractionation, which led to the isolation of the previously identified marine natural product, eusynstyelamide B (**1**). This *bis*-indole alkaloid was shown to display an IC_50_ of 5 µM in MDA-MB-231 cells. Moreover, **1** caused a strong cell cycle arrest in G2/M and induced apoptosis after 72 h treatment, making this molecule an attractive candidate for further mechanism of action studies.

## 1. Introduction

Currently, about 60% of anticancer drugs originate from natural products, their derivatives, or mimics [1]. Researchers have recognized that the incidence of biological activity in marine compounds is higher than in terrestrial-derived molecules [2,3]. The National Cancer Institute, USA, preclinical cytotoxicity screening demonstrated that approximately 1% of the tested marine samples showed antitumor potential compared to 0.1% of the tested terrestrial samples [4]. This 10-fold higher hit rate is linked to the greater chemical diversity associated with marine organisms, which is a product of the very different conditions found in the oceans, such as high salinity, high pressure, and relatively constant temperature [5]. Furthermore, it is thought that the immense competition for food and space challenges marine organisms to produce secondary metabolites for protection and survival [6].

Marine-derived compounds encompass a wide range of chemical classes, including terpenes, shikimates, polyketides, acetogenins, peptides, alkaloids, and many others [5]. It is estimated that there is US$563 billion–$5.69 trillion worth of anticancer marine-derived drugs still to be discovered [7]. Furthermore, only a small percentage of the world’s marine biodiversity has been examined for biodiscovery, and it has been predicted that 90.4%–92.6% of marine compounds still remain undiscovered [7]. Many marine compounds have generated interest for their cytotoxicity against multiple tumor types. The first marine-derived anticancer drug was ara-C (Cytosar-U^®^), inspired by the unusual nucleosides spongothymidine and spongouridine isolated from the sponge *Cryptotethya crypta* in the 1950s [8]. Among the marine invertebrates, ascidians have been a plentiful source of cytotoxic compounds. Analysis of the first six marine-derived drugs that have made anticancer clinical trials showed that three were isolated from ascidians [3]. The ascidian-derived compounds that have made clinical trials as antitumor agents are didemnin B [9], ecteinascidin 743 [10,11], and aplidine [12].

Breast cancer is the most common cancer in woman from developed countries [13]. For American women the chance of developing this type of cancer during a lifetime is about 12.4%, being 1.8% for women aged between 20–34 years, and 22.2% for women that are 45–54 years old [13]. It is also a major health problem for Australian woman, since it is the most common non-skin cancer, representing 28% of all reported cancers in females, and the second highest cause of cancer-related death in females [14]. Chemotherapeutics are usually used to treat patients in stage 2 or later stages of the disease, which have a higher risk of recurrence [15]. Different chemotherapeutics (anthracyclines, taxanes, alkylating agents, antimetabolites, *etc.*) alone or in combination are the main treatment for estrogen receptor (ER) negative (ER-negative) patients but are also used in combination with hormone blocking therapy for ER-positive patients [16].

In order to isolate new cytotoxic compounds with potentially new mechanisms of action in breast cancer, we screened 143 ascidian extracts in MDA-MB-231 cells (ER-negative) using a real time cell analyzer (RTCA) [17]. In addition, we conducted biological evaluation of the active extracts for effects on cell number, cell cycle distribution and cell morphology in order to prioritize the extracts for bioassay-guided fractionation. Following this approach, we isolated the previously reported natural product, eusynstyelamide B, which has never had cytotoxicity data reported.

## 2. Results

### 2.1. Time-Dependent Cell Response Analysis of Ascidian Extracts by RTCA

**Figure 1:**
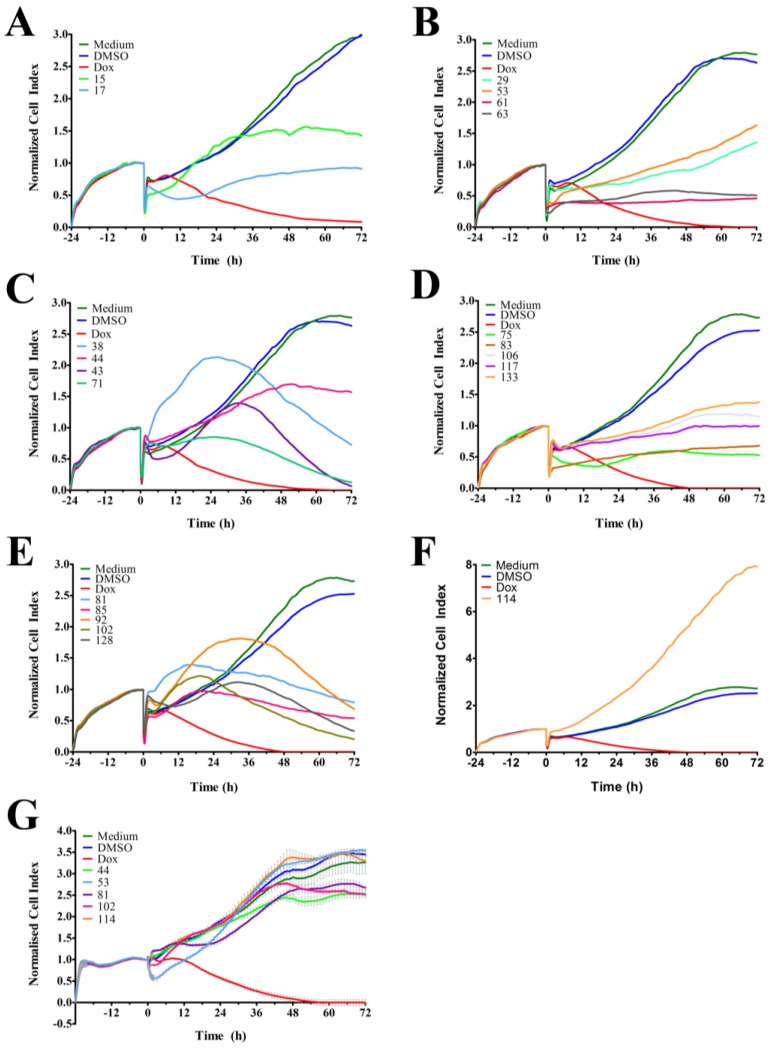
RTCA-generated time-dependent cell response profiles after treatment with ascidian extracts. (**A**–**F**) The real-time profiles display the seeding period (−24–0 h) followed by the treatment period (0–72 h) of MDA-MB-231 breast cancer cells. At 0 h, the indicated ascidian extracts were added at a final concentration of 1 µge/µL, and the cell index (CI) was measured every 2 h. As controls, cells were treated with DMSO (0.1%), doxorubicin (Dox, 10 µM), or remained untreated (medium); (**G**) Representative RTCA profiles of non-malignant NFF cells treated with the indicated ascidian extracts and controls.

The RTCA-generated time-dependent cell response profile (TCRP) is a composite measure of how a bioactive compound affects the growth rate, morphology and adhesive characteristics of a cell culture. A previous study tested 2000 small molecule compounds by RTCA and demonstrated that compounds with similar activity produced similar TCRPs [18]. This study showed that inhibitors of G protein-coupled receptors and calcium signaling, nucleotide and DNA synthesis, tubulin polymerization, nuclear hormone receptors, and protein synthesis could be classified based on their unique TCRP [18]. In our studies, we tested 143 ascidian extracts in MDA-MB-231 cells by RTCA and identified 21 extracts (Table 1) that affected the cell index (CI), which is a quantitative and composite measure of the overall state of the cells in an electrode-containing well, and generated a TCRP that was profoundly different to the DMSO control (Figure 1 and Table 1). Twenty of these extracts decreased the CI (Figure 1A–E), while one extract increased the CI (Figure 1F) after 72 h of treatment when compared to DMSO control. Extracts 38, 43, 71, 81, 85, 92, 102, and 128 caused a bell-shaped TCRP, which was characterized by an initial rise of the CI followed by a decrease of the CI, the latter suggesting a cytotoxic response (Figure 1C,E) [18]. Interestingly, the onset of cytotoxicity (peak in CI) varied among these extracts and clustered at 18, 24, and 30 h post treatment. Conversely, extracts 17 and 75 caused the CI to initially decline with the lowest point at 12–14 h post treatment followed by a recovery and increase of the CI (Figure 1A,D). The remaining active extracts induced a cytostatic effect (extracts 15, 44, 61, 63, and 106) or caused a slower rise of the CI (extracts 29, 53, 83, 117, and 133) (Figure 1A–D). Extract 114 was unique among the active extracts and caused the CI to substantially increase when compared to the control (Figure 1D).

Next, we tested the 21 active ascidian extracts in the non-malignant neonatal foreskin fibroblast (NFF) cell line to assess anti-neoplastic selectivity. Sixteen extracts (15, 17, 29, 38, 43, 61, 63, 71, 75, 83, 85, 92, 106, 117, 128, and 133) displayed severe adverse effects in NFF cells (data not shown). In contrast, extracts 53 (decrease of CI in MDA-MB-231 cells) and 114 (increase of CI in MDA-MB-231 cells) displayed TCRPs that were similar to the controls (medium and DMSO, Figure 1G), suggesting that they did not affect NFF cells and were potentially selective in malignant cells. Furthermore, extracts 44, 81, and 102 showed only modest effects on the TCRP of NFF cells when compared to MDA-MB-231 cells, suggesting that they possess anti-neoplastic selectivity.

In order to prioritize the active ascidian extracts identified by RTCA for bioassay-guided fractionation, we investigated different biological aspects like cell number, morphology and cell cycle distribution of MDA-MB-231 cells treated for 24 h. Notably, these tests individually address cellular characteristics, which comprise the composite figure of the CI measured by RTCA.

**Table 1:** Taxonomic details for the 21 most active ascidian extracts that displayed cytotoxicity in the breast cancer cell line MDA-MB-231. All ascidian samples were collected from the Great Barrier Reef (Queensland, Australia) and were identified by the Queensland Museum.

Extract Number	Species
15	*Polysyncraton millepore*
17	*Trididemnum cf. cerebriforme*
29	*Trididemnum pigmentatum*
38	*Polysyncraton echinatum*
43	*Leptoclinides kingi*
44	*Leptoclinides durus*
53	*Trididemnum sibogae*
61	*Lissoclinum badium*
63	*Polysyncraton pseudorugosum*
71	*Leptoclinides dubius*
75	*Polysyncraton pseudorugosum*
81	*Leptoclinides durus*
83	*Leptoclinides dubius*
85	*Lissoclinum fungium*
92	*Leptoclinides kingi*
102	*Didemnum candidum*
106	*Didemnum multispirale*
114	*Didemnum candidum*
117	*Didemnum membranaceum*
128	*Didemnum membranaceum*
133	*Didemnum guttatum*

### 2.2. Cell Count after Treatment with Ascidian Extracts

**Figure 2:**
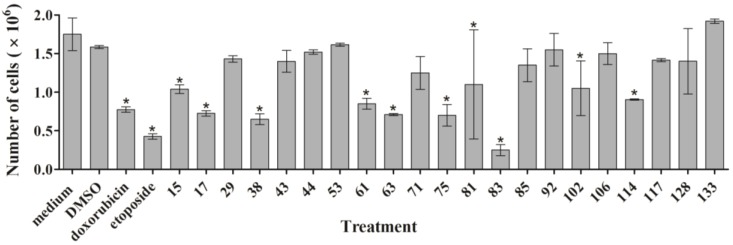
MDA-MB-231 cells were treated for 24 h with 1 µge/µL of the indicated ascidian extracts. Cells were harvested, stained with Trypan Blue, and live cells were counted. The results are shown as mean ± SD (*n* = 3). Statistically significant results (*p* < 0.05) are marked with an asterisk.

First, we measured the effect of the active ascidian extracts on the growth of MDA-MB-231 cells by counting the total number of cells after treatment for 24 h. Extracts 15, 17, 38, 61, 63, 75, 81, 83, 102, and 114 significantly reduced the cell number when compared to the DMSO control (Figure 2), demonstrating that they negatively affected proliferation.

### 2.3. Analysis of Cell Morphology by Microscopy

**Figure 3:**
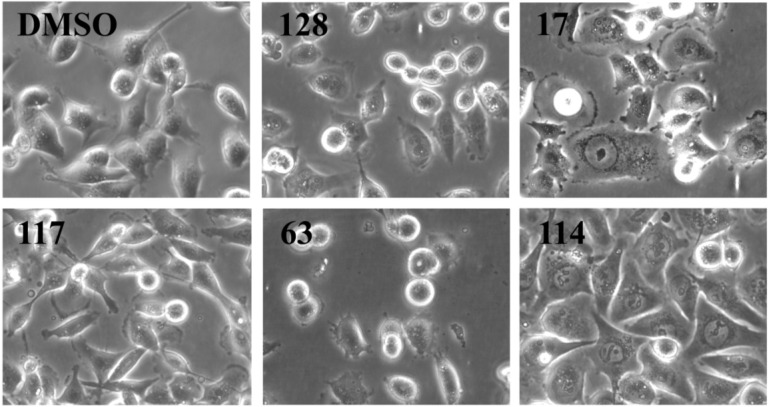
Morphology analysis of MDA-MB-231 cells treated for 24 h with the indicated ascidian extracts (1 µge/µL). As controls, cells were treated with DMSO (0.1%). Part of the original images (Supplementary Figure S1) were zoomed in and presented below. Images were obtained with an Olympus IX70 microscope using a 10× objective.

We analyzed cell morphology of MDA-MB-231 cells treated for 24 h with all 21 active ascidian extracts by phase contrast microscopy (Figure 3 and Supplementary Figure S1). Cells treated with extracts 43, 128 and 133 displayed a similar morphology when compared to the negative controls (DMSO and medium), with round semi-attached cells without processes and flat cells with established cell-cell contacts. Extracts 15, 17, 83, and 106 induced morphological changes like cell shrinkage, rounding up, loss of processes and cell-cell contacts. In addition, cells treated with extracts 15 and 17 presented membrane blebbing, a typical sign associated with cell death through apoptosis [19], which was also observed with doxorubicin treatment. Extracts 29, 38, 44, 85, 92, 102, and 117 appeared to fasten the process of attachment, as indicated by a reduced number of round semi-attached cells and an increase in eccentricity and cell-cell contacts. Conversely, extracts 53, 63, and 75 seemed to cause cells to detach. Extracts 61, 71, 81, and 114 produced a phenotype where cells were flat and enlarged.

### 2.4. Cell Cycle Studies

**Figure 4:**
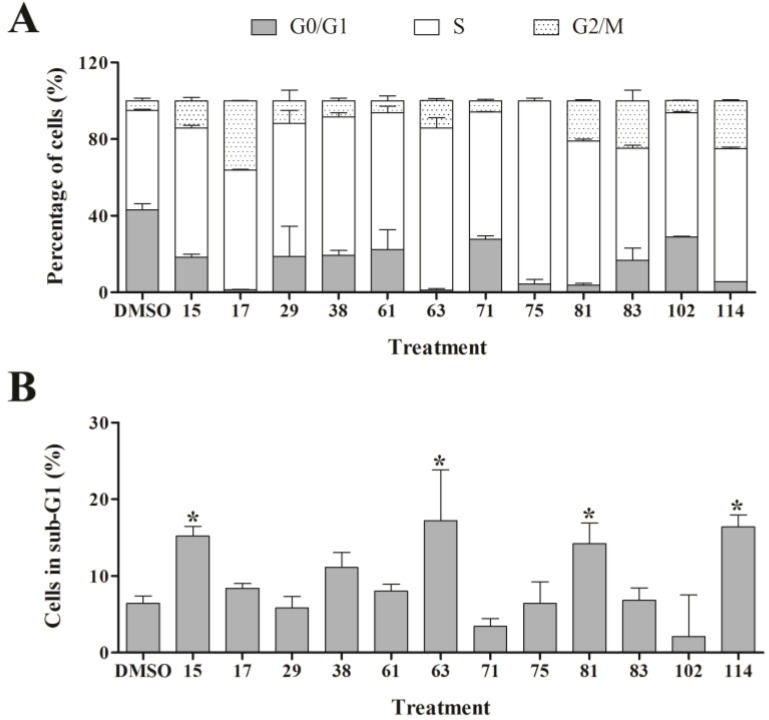
Cell cycle analysis of MDA-MB-231 cells treated with bioactive ascidian extracts. MDA-MB-231 cells were treated with the indicated bioactive ascidian extracts for 24 h and DNA content was measured by flow cytometry and quantified with ModFit LT 3.3 software. As control, cells were treated with DMSO (0.1%). Only the results of the extracts that arrested the breast cancer cells are shown below. (**A**) Cell cycle distribution of cells in G0/G1, S and G2/M phase of the cell cycle (mean ± SD, *n* = 3). Statistical information can be found in Supplementary Data (Supplementary Table S1); (**B**) Dead cells with hypodiploid DNA content (subG1) (mean ± SD, *n* = 3). Statistically significant results (*p* < 0.05) are highlighted with an asterisk.

In order to assess the effect of the active ascidian extracts on the cell cycle of MDA-MB-231 cells, we performed flow cytometry and measured the DNA content. Interestingly, more than half of the 21 ascidian extracts selected by RTCA affected the cell cycle distribution of MDA-MB-231 cells when compared to control (0.1% DMSO, Figure 4 and Supplementary Table S1). The majority of cell cycle modulating extracts caused an increase of the number of cells in the S and G2/M phases, and a corresponding sharp drop in the number of cells in G0/G1. Of particular interest was extract 75, which displayed an almost universal S phase arrest (95.7%). Furthermore, extracts 17, 81, 83, and 25 increased the G2/M cell population by 4- to 7-fold when compared to control, suggesting that these extracts induced a cell cycle arrest in G2/M. Extracts 15, 63, 81 and 114 provoked a significant increase in the number of cells with hypo-diploid DNA content (sub-G1) which is caused by DNA fragmentation, a late stage process of cell death induced through apoptosis or necrosis (Figure 4).

### 2.5. Prioritization, Extraction and Bioassay-Guided Fractionation of Ascidian Extract 114 (Didemnum candidum)

**Figure 5:**
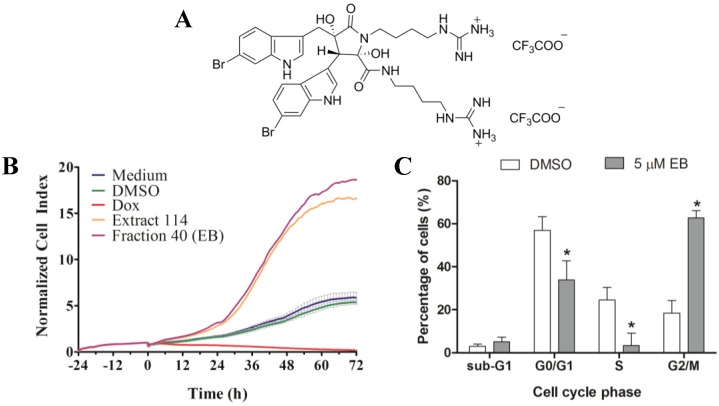
(**A**) Structure of the *bis*-TFA salt of eusynstyelamide B (EB, **1**); (**B**) RTCA profile of MDA-MB-231 cells treated for 72 h with EB (**1**) and ascidian extract 114. As controls, cells were treated with DMSO (0.1%), doxorubicin (Dox, 10 µM), or remained untreated (medium); (**C**) Cells were treated for 72 h with 5 µM of EB (**1**) and cell cycle distribution (G0/G1, S and G2/M phase), and cell death (subG1) were measured by flow cytometry and quantified with ModFit LT 3.3 software (mean ± SD, *n* = 3). Statistically significant results (*p* < 0.05) are highlighted with an asterisk.

The RTCA-based TCRP of extract 114 suggested an apparently positive effect on MDA-MB-231 cells, as indicated by the greater CI relative to the DMSO and medium controls (Figure 1F). However, subsequent analysis of proliferation, morphology and cell cycle revealed that extract 114 inhibited cell growth, increased cell size, induced a G2/M cell cycle arrest, and significantly increased the number of dead cells (sub-G1), demonstrating that extract 114 was cytotoxic in MDA-MB-231 cells. Hence, it was prioritized as the first sample from these studies to be subjected to bioassay-guided fractionation. The freeze-dried and ground ascidian, *Didemnum candidum*, was sequentially and exhaustively extracted with *n*-hexane, CH_2_Cl_2_:MeOH (4:1) and MeOH. The *n*-hexane extract was discarded, while all CH_2_Cl_2_/MeOH extracts were combined then subjected to semi-preparative C_18_ HPLC (MeOH/H_2_O/0.1% TFA). The resulting HPLC fractions were tested in MDA-MB-231 cells using the RTCA, which identified two active fractions (Figure 5B and Supplementary Figure S2) that both contained the same compound (**1**, 3.5 mg, 0.035% dry wt). Analysis of the 1D (Supplementary Figures S3 and S4) and 2D NMR (Supplementary Figures S5–S7), [α]_D_, CD and MS data for compound **1** in conjunction with comparison of literature data identified this compound as the *bis*-TFA salt of the previously reported *bis*-indole alkaloid, eusynstyelamide B (EB, **1**) [20,21].

### 2.6. Determination of IC_50_ for Eusynstyelamide B (**1**)

Analysis of MDA-MB-231 cells treated for 72 h with serial dilutions of **1** by AlamarBlue^®^ assay revealed an IC_50_ of 4.95 µM with a 95% CI varying from 3.8 to 6.3 µM (Supplementary Figure S8).

### 2.7. Validation of **1** by RTCA and Cell Cycle Analysis

As shown above, extract 114, from which **1** was isolated, substantially increased the CI when compared to control. However, it arrested MDA-MB-231 cells in the G2/M phase of the cell cycle and increased the number of dead cells after 24 h of treatment. In order to verify if **1** was responsible for the activity observed, MDA-MB-231 cells were treated with 5 µM of compound **1** and monitored by RTCA. As shown in Figure 5B, **1** generated a time-dependent cell response profile, which was similar to that of the crude active extract 114. Cell cycle analysis of MDA-MB-231 cells treated with 5 µM of compound **1** for 72 h revealed a significant arrest in the G2/M phase (Figure 5C) with a corresponding significant decrease of the G0/G1 and S phase cell populations. However, **1** did not significantly increase the number of dead cells (sub-G1).

### 2.8. Mode of Cell Death Studies

**Figure 6:**
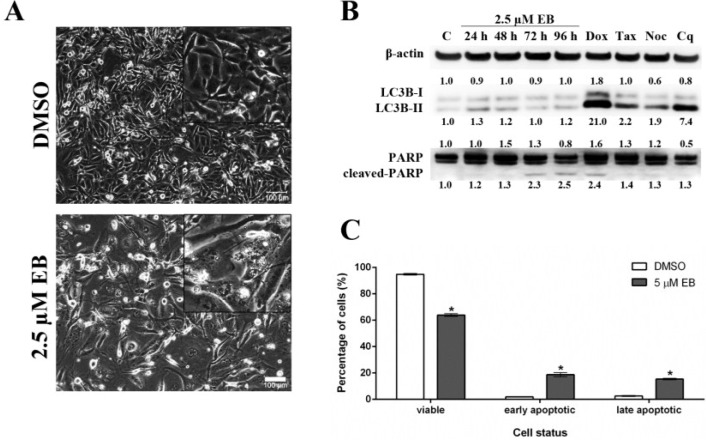
Eusynstyelamide B (EB, **1**) induces cell death in MDA-MB-231 cells through apoptosis. (**A**) MDA-MB-231 cells were treated with 2.5 µM EB or 0.1% DMSO for 96 h and imaged using an Olympus IX70 microscope (10× objective, scale bar = 100 µm); (**B**) Cells were treated with 2.5 µM EB for the indicated times or 0.1% DMSO for 96 h (C). As positive controls for apoptosis (PARP cleavage), cell were treated with 1 µM doxorubicin (Dox) for 48 h, 2 nM taxol (Tax) for 24 h or 83 nM nocodazole (Noc) for 24 h. As a positive control for autophagy (LC3B-II), cell were treated with 25 µM chloroquine (Cq) for 48 h. Expression of the indicated proteins was assessed by Western blotting, normalized against the level of β-actin, and is expressed in fold-change relative to control (C); (**C**) MDA-MB-231 cells were treated with 5 µM EB or 0.1% DMSO for 96 h, stained with PI and Annexin V-FITC, and the number of viable, early apoptotic and late apoptotic/necrotic cells was quantified by flow cytometry (mean ± SD, *n* = 3, * *p* < 0.05).

As shown above, treatment with eusynstyelamide B (EB, **1**) caused a mild increase in the number of MDA-MB-231 cells in sub-G1, which indicates DNA fragmentation and cell death; however, the increase in cell death was not statistically significant when cells were treated for 72 h (Figure 5C). When MDA-MB-231 cells were treated for 96 h with **1**, phase contrast microscopy (Figure 6A) revealed morphological changes which are characteristic for cell death by apoptosis, e.g., loss of cell-cell contacts, cell shrinkage and membrane blebbing. Two modes of cell death, autophagy and apoptosis, were analyzed by Western blotting. During autophagy, cleavage of the protein light chain 3 (LC3B) at the carboxyl terminus yields the cytosolic LC3B-I form. LC3B-I is converted to LC3B-II through lipidation. MDA-MB-231 cells treated with 2.5 µM of **1** for up to 96 h did not increase the formation of LC3B-II (Figure 6B), demonstrating that **1** did not induce autophagy. PARP (116 kDa) is involved in DNA repair and is the main cleavage target of caspase-3. Cleaved PARP (89 kDa) is a well-known marker of apoptosis. MDA-MB-231 cells treated with EB for 72 h and 96 h showed increased levels of cleaved PARP, indicating that **1** induced apoptosis (Figure 6B). In addition, flow cytometry of Annexin V/PI-stained MDA-MB-231 cells demonstrated that **1** significantly reduced the number of viable cells and induced a significant increase in the number of cells in early apoptosis and late apoptosis/necrosis after 96 h of treatment (Figure 6C).

## 3. Experimental Section

### 3.1. General

NMR spectra were recorded at 30 °C on a Varian 600 MHz spectrometer equipped with a triple resonance cold probe. The ^1^H and ^13^C chemical shifts were referenced to the solvent peak for DMSO-*d*_6_ at δ_H_ 2.49 and δ_C_ 39.5, respectively. LRESIMS were recorded on a Applied Biosystems Mariner Biospectrometry TOF workstation using either negative or positive electrospray ionization, and a mobile phase of 1:1 MeOH:H_2_O. The [α]_D_ was measured on a JASCO P-1020 polarimeter The CD spectrum was recorded on a JASCO J-715 spectopolarimeter. Phenomenex SPE polypropylene cartridges (10 × 50 mm) with a 20 µm nylon frit inserted were used for small-scale extractions of the 143 ascidian samples. A Bio-line orbital shaker was used for the large-scale ascidian extraction. Alltech Davisil 30–40 µm 60 Å C_18_ bonded silica was used to pre-adsorb the ascidian extract prior to HPLC separation. An Alltech stainless steel guard cartridge (10 × 30 mm) was used for packing the pre-adsorbed material. A Waters 600 pump equipped with a Waters 996 PDA detector and a Waters 717 autosampler were used for HPLC separations. LC/MS analysis was performed on a Waters Alliance system (2790) using a Phenomenex Luna C_18_ 3 µm 100 Å column (4.6 × 50 mm). A C_18_ Betasil 5 µm 143 Å column (21.2 mm × 150 mm) was used for semi-preparative HPLC separation. All solvents used for chromatography, [α]_D_ CD, and MS were Lab-Scan HPLC grade, and the H_2_O was Millipore Milli-Q PF filtered.

### 3.2. Ascidian Material

A total of 143 ascidian specimens (Nature Bank Biota Library (http://www.nature-bank.com.au)) belonging to the family Didemnidae were selected for the generation of the extract library. The selection was based on the amount of material available and exclusion of species that have been extensively studied [22]. All marine samples were collected from the Great Barrier Reef (Queensland, Australia) and taxonomically identified by the Queensland Museum. Samples were kept frozen prior to freeze-drying and extraction. Voucher samples have been lodged at the Queensland Museum, Brisbane, Australia.

### 3.3. Generation of the Ascidian Extract Library

A small amount (300 mg) of freeze-dried and ground sample from each of the 143 didemnids was packed into a SPE cartridge, and extracted with CH_2_Cl_2_ (8 mL), then MeOH (8 mL). Both extracts were combined, dried down, and resuspended in DMSO (300 μL) to yield 143 extracts at a concentration of 1000 µge/µL. The concentration units are related to the extract derived from the dry and ground ascidian sample, which in this instance was 300 mg equivalents, *i.e.*, mge.

### 3.4. Cell Culture

MDA-MB-231 (ATCC) and NFF (Dusan Zencak, Eskitis Institute) cells were cultured in DMEM medium (Invitrogen, Carlsbad, CA, USA) supplemented with 10% (v/v) heat-inactivated FBS (Invitrogen, Carlsbad, CA, USA) at 37 °C in a 5% CO_2_ humidified environment.

### 3.5. Real Time Cell Analysis of the Ascidian Extract Library

The 143 ascidian extracts were screened for cytotoxicity in MDA-MB-231 breast cancer cells using RTCA xCELLigence System (Roche, Basel. Switzerland). First, 100 µL of complete medium were added to each well for background measurement. Then, 4000 MDA-MB-231 cells/well or 2000 cells/well of the non-malignant neonatal foreskin fibroblast cell line NFF were seeded in a 96-well E-plate^®^. The E-plate^®^ was incubated at room temperature for 30 min and placed on the reader in the incubator for continuous recording of impedance to compute the cell index (CI). After 24 h, the cells were treated with 0.1% (v/v) of each ascidian extract (1 µge/µL), 0.1% (v/v) DMSO (Sigma-Aldrich, St. Louis, MO, USA) or 10 µM of doxorubicin (Sigma-Aldrich, St. Louis, MO, USA). Each extract was analyzed in two independent experiments, and extracts were considered active only if both experiments revealed an effect on the CI. The impedance-based CI was quantified by RTCA software program version 1.2.1 (Roche, Basel. Switzerland), and normalized according to the last time point prior the start of treatment (*t* = 0 h). From this data, TCRP were generated with GraphPad Prism 5 (GraphPad Software, La Jolla, CA, USA).

### 3.6. Cell Counting of the Ascidian Extract Library

Each well of a 6-well plate was seeded with 0.3 × 10^6^ cells. After 24 h, the growth medium was substituted by 2 mL of fresh medium containing 1 µge/µL of the ascidian extracts, 0.1% (v/v) DMSO (Sigma-Aldrich, St. Louis, MO, USA), 10 µM doxorubicin (Sigma-Aldrich, St. Louis, MO, USA), 25 µM etoposide (Sigma-Aldrich, St. Louis, MO, USA) or culture medium, and the plates were incubated at 37 °C in 5% CO_2_. After 24 h, cells were harvested and an aliquot was stained with Trypan Blue Solution (0.4%, Invitrogen, Carlsbad, CA, USA), followed by cell counting using a hemocytometer (Neubauer Improved, Sigma-Aldrich, St. Louis, MO, USA). The experiment was carried out in duplicate, and results were expressed as mean ± SD and analyzed using Student’s *t*-test. Results with *p* < 0.05 were considered statistically significant.

### 3.7. Cell Morphology Analysis by Phase-Contrast Microscopy

Each well of a 6-well plate was seeded with 0.3 × 10^6^ cells. After 24 h, the cells were treated as described above and incubated for 24 h. Alternatively, cells were treated with 2.5 µM EB or 0.1% DMSO for 96 h. The cells were then photographed using an objective with 20× magnification (Olympus IX70, Olympus, Tokyo, Japan).

### 3.8. Cell Cycle Studies of the 21 Active Ascidian Extracts

The 21 ascidian extracts that were classified as active by RTCA screening were further evaluated by cell cycle analysis. MDA-MB-231 cells were seeded and treated as above for 24 h. Alternatively, MDA-MB-231 cells were treated for 72 h with 5 µM of compound **1**. Cells were harvested and washed twice with ice cold DPBS (Invitrogen, Carlsbad, CA, USA). After overnight fixation with 70% EtOH (v/v) in DPBS at −20 °C, cells were centrifuged and resuspended in 1 mL of 30 µg/mL PI (w/v) (Sigma-Aldrich, St. Louis, MO, USA) and 20 µg/mL RNAse A (w/v) (Sigma-Aldrich, St. Louis, MO, USA) in DPBS (Invitrogen, Carlsbad, CA, Country), and incubated overnight at 4 °C in the dark. DNA content was determined by flow cytometry using a BD FACSCanto^™^ Flow Cytometer (BD Biosciences, San Jose, CA, USA). A minimum of 10,000 events were recorded per sample. The percentage of cells in each stage of the cell cycle was calculated using ModFit 3.3 software (Verity Software, Topsham, ME, USA). The results of three independent experiments were expressed as mean ± SD and statistically analyzed using one-way ANOVA. Results with *p* < 0.05 were considered significant.

### 3.9. Large-Scale Extraction of Didemnum candidum (Ascidian Code: 114)

The freeze-dried and ground raw material (10 g) of *Didemnum candidum* (ascidian code: 114) was poured into a separate conical flask (1 L); *n*-hexane (250 mL) was added and the flask was shaken at 200 rpm for 2 h. The *n*-hexane extract was filtered under gravity, and discarded. CH_2_Cl_2_:MeOH (4:1, 250 mL) was then added to the de-fatted ascidian sample in the conical flask and shaken at 200 rpm for 2 h. The resulting extract was filtered under gravity, and set aside. MeOH (250 mL) was added to the ascidian sample, and the flask shaken at 200 rpm for 2 h before filtration. Another volume of MeOH (250 mL) was then added and the MeOH/ascidian mixture was shaken for a further 16 h at 200 rpm, followed by gravity filtration. All CH_2_Cl_2_/MeOH extracts were combined and dried under reduced pressure to afford a crude extract (536 mg) that was stored at 4 °C prior to bioassay-guided fractionation studies.

### 3.10. Bioassay-Guided Fractionation of the Extract from *Didemnum candidum* (Ascidian Code: 114)

The CH_2_Cl_2_/MeOH extract (536 mg) was pre-adsorbed onto C_18_-bonded silica (~1 g) then packed into an Alltech stainless steel guard cartridge (10 × 30 mm). This cartridge was attached to a semi-preparative C_18_ Betasil HPLC column (21.2 mm × 150 mm) then subjected to isocratic solvent conditions of 10% MeOH (0.1% TFA)/90% H_2_O (0.1% TFA) for the first 10 min, followed by a linear gradient to MeOH (0.1% TFA) in 40 min, then isocratic conditions of MeOH (0.1% TFA) for 10 min, all at a flow rate of 9 mL/min. Sixty fractions (60 × 1 min) were collected from the start of the HPLC run. RTCA analysis of the 60 fractions determined that the bioactive material eluted in fraction 40 and 41 (time = 40–41 min). 1H NMR and MS analysis of these two fractions revealed that they both contained the same compound, and were, thus, combined. Analysis of the 1D and 2D NMR, [α]_D_, CD and MS data for this compound in conjunction with comparison of literature data identified this compound as the *bis*-TFA salt of the previously reported *bis*-indole alkaloid, eusynstyelamide B (**1**, 3.5 mg, 0.035% dry wt) [20,21].

### 3.11. Determination of the IC_50_ of Eusynstyelamide B (**1**)

The IC_50_ of **1** was determined using the AlamarBlue^®^ assay (Invitrogen, Carlsbad, CA, USA). MDA-MB-231 cells were seeded into a polystyrene black clear-bottomed 96-well plate at a density of 4000 cells/well. After 24 h, the cells were treated with different concentrations of **1** (0–100 µM) and incubated for 72 h. AlamarBlue^®^ assay was performed according to the manufacturer’s instruction (Invitrogen, Carlsbad, CA, USA). Average values of triplicates were calculated after background correction. The IC_50_ was calculated by non-liner regression analysis with GraphPad Prism 5 (GraphPad Software, La Jolla, CA, USA).

### 3.12. Western Blotting Experiments Using Eusynstyelamide B (**1**)

MDA-MB-231 cells (0.1 × 10^6^) were seeded in a 6 well/plate and treated with 5 µM of compound **1** for the indicated times. As positive controls, cells were treated with doxorubicin (1 µM, 48 h), etoposide (25 µM, 24 h), chloroquine (25 µM, 48 h), taxol (2 nM, 24 h), or nocodazole (83 nM, 24 h) which were purchased from Sigma Aldrich (Sigma-Aldrich, St. Louis, MO, USA). 0.1% DMSO was used as vehicle control. At the end of the treatment, cells were harvested and lysed with lysis buffer containing protease inhibitor cocktail (Roche, Basel, Switzerland). Protein concentration was determined with a bicinchoninic protein assay kit (BCA assay, Thermo Fisher Scientific, Waltham, MA, USA). Thirty micrograms of protein lysates per sample were loaded onto a NuPAGE^®^ 4%–12% Bis-Tris Gel (Invitrogen, Carlsbad, CA, USA) and separated by electrophoresis for 90 min at 135 V. Proteins were transferred to a nitrocellulose membrane by wet transfer for 1 h at 100 V. Primary antibodies were used as recommended by the manufacturer; PARP antibody (#9542, Cell Signaling, Danvers, MA, USA), LC3B (#3868, Cell Signaling, Danvers, MA, USA) and beta-actin (04-1116, Merck Millipore, Billerica, MA, USA). Membranes were developed with the appropriate horseradish peroxidase-conjugated secondary antibody (GE Healthcare, Pittsburgh, PA, USA) and visualized with a chemiluminescence reaction system (Immobilon Western Chemiluminescent HRP Substrate, Merck Millipore, Billerica, MA, USA) and documented on a ChemiDoc XRS system (Bio-Rad, Hercules, CA, USA). Proteins were quantified using Image Lab™ software (Bio-Rad, Hercules, CA, USA), normalized according to the respective beta-actin levels, and were expressed as fold-change relative to the control treatment.

### 3.13. Annexin V Assay Using Eusynstyelamide B (**1**)

The assay was carried out according to the manufacturer’s instruction (BioVision, Milpitas, CA, USA). Briefly, MDA-MB-231 (0.1 × 10^6^) cells were seeded in a 6 well/plate and treated with 5 µM EB or 0.1% DMSO. After 96 h, cells were harvested, washed twice with cold DPBS (Invitrogen) and counted. 1 × 10^5^ cells were resuspended in 500 µL 1× binding buffer, containing 1 µL (0.15 mg/mL) of FITC-conjugated Annexin V (BioVision, Milpitas, CA, USA) and 1 µL of propidium iodide (PI, 1 mg/mL, Sigma-Aldrich, St. Louis, MO, USA) and incubated at room temperature for 20 min in the dark. After electronic compensation, fluorescence of 20,000 cells were measured in the PI and FITC channels by flow cytometry (FACSCanto, BD Biosciences, San Jose, CA, USA), and data were analyzed with FASCDiva software (BD Biosciences, San Jose, CA, USA). Double negative-stained cells were counted as viable cells, PI-negative/FITC-positive cells were counted as early apoptotic cells and double positive-stained cells were counted as late apoptotic/necrotic cells. Results were analyzed using one-way ANOVA (*p* < 0.05).

## 4. Discussion

In order to identify and isolate new cytotoxic ascidian-derived compounds, 143 ascidian extracts were generated then subsequently screened in MDA-MB-231 breast cancer cells. Out of 143 ascidian extracts, we identified 21 extracts that affected the growth of MDA-MB-231 cells when tested by RTCA (Figure 1). We observed substantial differences in the TCRPs among the active extracts. A few extracts caused an effect immediately after cells were treated, and both positive and negative effects on the CI were observed. However, the majority of active extracts required several hours or days to cause a divergence of the CI when compared to the controls. This time-dependent effect could have been due to the necessity of metabolic activation of the compound, accumulation of the compound, compound membrane solubility/uptake across membranes, accumulation of toxic intermediates, or end-product starvation among other reasons [23]. In addition to the identification of active extracts, the RTCA-based TCRPs provided important information, such as optimal treatment period for subsequent end-point validation assays. A previous study, which tested 2000 small molecule compounds by RTCA, showed that compounds with a similar mode of action produced a similar TCRP, thereby providing predictive mechanistic information [18]. However, the interpretation of extract-based TCRPs for the purpose of mechanism-of-action predictions might be limited due to the complexity of the active ascidian extracts. We did however observed that the TCRPs of most of the 21 active ascidian extracts fell into two previously reported and defined categories (Figure 1) [18]. The first category included bell shaped TCRPs, which has been found to be associated with inhibitors interfering with DNA synthesis, transcription and translation, such as 5-fluorouracil, etoposide, and MG132 [18]. The second category, constituted TCRPs that showed an initial decline of the CI followed by a recovery and increase of the CI, which is typical for mitotic inhibitors, such as paclitaxel, nocodazole and colchicine [18].

As outlined above, the RTCA measurement represents a composite figure of how a bioactive compound affects cell growth, morphology and adhesive characteristics of a cell culture. For validation purposes, we sought to address two of these cellular properties (growth and morphology) individually by performing cell count (Figure 2) and phase contrast microscopy (Figure 3, Supplementary Figure S1) after treatment of MDA-MB-231 cells for 24 h. Cell counting demonstrated that nine extracts (15, 17, 38, 61, 63, 75, 83, 102, and 114) significantly reduced the number of cells compared to the control (Figure 2). The RTCA data suggested that a longer incubation period might have facilitated more extracts reaching significance in reducing the cell number. Notably, none of the extracts increased the cell number after 24 h of treatment as implied by the RTCA data of extracts 38, 81, 92, 102, and 114, suggesting that changes to cell morphology and/or adherence caused the elevated CI. This observation was best exemplified by extracts 38 and 114 and highlighted the sensitivity of RTCA to changes in these cellular characteristics. Indeed, microscopy suggested that these extracts improved the adherence of MDA-MB-231 cells (38, 92, and 102), with fewer rounded and semi-attached cells present, and/or increased the cell size (81 and 114) when compared to control (Figure 3, Supplementary Figure S1). Furthermore, it confirmed the reduced cell numbers caused by extracts 15 and 17 (Figure 2) by showing the typical signs of apoptosis, *i.e.*, loss of cell-cell contacts, cell shrinkage and membrane blebbing (Figure 3). In summary, cell counting and microscopy were two relative easy and efficient assays that helped to de-convolute and interpret the complex RTCA data and facilitated the prioritization of extracts for bioassay-guided fractionation. Additional functional characterization of the extracts by cell cycle analysis of MDA-MB-231 cells (Figure 4, Supplementary Table S1) revealed that the majority of extracts caused arrest or delay of S phase (DNA synthesis) and G2/M phase (G2/mitosis), with extracts 15, 63, 81, and 114 leading to significant DNA fragmentation and cell death (subG1), possibly by inducing apoptosis. The absence of a significant subG1 cell population after treatment with extract 83, which caused the highest reduction in cell number among the extracts and a strong and immediate negative impact on the CI in RTCA suggested that pathways other than apoptosis or anoikis (e.g., autophagy) induced cell death. Interestingly, none of the extracts induced a G0/G1 cell cycle arrest. Consistent with our cell cycle results, previous studies have shown that ascidians are a rich source of DNA intercalating compounds and topoisomerase inhibitors [24,25]. Both classes of compounds cause DNA damage which can lead to S phase or G2/M arrest and are usually cytotoxic to different cell lines. RTCA screening of the 21 extracts in the non-malignant fibroblast cell line NFF revealed a relatively high level of general toxicity among the extracts, reinforcing the hypothesis of the presence of the aforementioned classes of compounds in the ascidian extracts. Only extracts 44, 53, 81, 102 and 114 were less toxic or cytostatic at the same dose in NFF cells when compared to the breast cancer cell line (Figure 1G), suggesting some anti-cancer specificity.

The following criteria were used to prioritize the ascidian extracts for bioassay-guided fractionation: cell cycle effects, specific and general cytotoxicity, cell morphology, and logistic reasons, such as the amount of ascidian material available in Nature Bank (http://www.nature-bank.com.au). Only two extracts, 102 and 114, reduced the number of cells after treatment, caused cell cycle arrest, altered breast cancer cell morphology, and did not display cytotoxicity in NFF cells. Because of the larger amount of ascidian material available in NatureBank, extract 114 was selected for bioassay-guided fractionation, and yielded the known alkaloid dimer, eusynstyelamide B (EB, **1**, Supplementary Figures S3–S7). Compound **1** was previously isolated from the Great Barrier Reef ascidian *Eusynstyela latericus* [20]. It is the stereoisomer of eusynstyelamide A, which was first isolated from the ascidian *E. misakiensis*, but whose chemical structure was initially misassigned [20,26]. Recently, the total synthesis of eusynstyelamide A was accomplished in six steps in 13% overall yield from 6-bromoindole, methyl glycidate, and Boc-protected agmatine [27]. Motti *et al.* suggested that **1** might be the result of the dimerization of two modified dipeptides formed from α-keto acid, derived from tryptophan, and agmatine by decarboxylation of arginine [20]. Compound **1** has been shown to have mild antibacterial activity towards *Staphylococcus aureus* (IC_50_ 6.5 µM), and inhibited the C4 plant regulatory enzyme pyruvate phosphate dikinase (IC_50_ 20 µM) [20]. The enantiomer of **1**, ent-eusynstyelamide B, isolated from the Arctic bryozoan *Tegella* cf. *spitzbergensis* was found to display anti-bacterial activity in *S. aureus* (MIC 7.95 µM) and *Escherichia coli* (MIC 15.9 µM) and was weakly active in *Candida albicans* (MIC 127.2 µM) [21]. The presence of stereoisomers and enantiomers of **1** in different phyla localized in different climatic zones suggests that the biosynthesis of this particular molecule may be performed by different microorganisms associated with the marine macro-organism [28,29]. Despite efforts by several research groups to determine the absolute configuration of the eusynstyelamide series, this currently remains unassigned. [20,21].

Previous work has found by colorimetric sulforhodamine B assay that **1** was not cytotoxic in the cancer cell lines MCF-7 (breast), SF-268 (CNS), and H-460 (lung) when tested up to 32 mM [20]. Our discovery that **1** is cytotoxic in MDA-MB-231 cells, as shown by four different methods, might be due to differences in methodology and characteristics of the cell lines used. Our studies further revealed that **1** induced cell death of MDA-MB-231 cells through apoptosis. Interestingly, **1** has been reported to inhibit neural nitric oxide synthase (NOS1) with an IC_50_ of 4.3 µM. NOS1 as well as its isoforms, inducible NOS (NOS2) and endothelial NOS (NOS3) are expressed in MDA-MB-231 cells [30]. Thus, the nitric oxide synthase family could be a potential target of **1** in these cells.

## 5. Conclusions

In summary, the use of RTCA along with cell cycle analysis and microscopy studies provide an efficient way to prioritize ascidian extracts for further investigation of the cytotoxic compounds. The cytotoxicity screening in MDA-MB-231 cells and bioassay-guided fractionation of a prioritized ascidian extract identified the cytotoxic compound, eusynstyelamide B (**1**) (IC_50_ 5.0 µM). This is the first report of cytotoxic activity for this rare marine natural product structure class in a cancer cell line. Due to the structure uniqueness of **1**, this molecule is an attractive candidate for more detailed mechanism of action studies.

## Supplementary Files

Supplementary File 1
