# Supplementary material for: Identification of Eusynstyelamide B as a Potent Cell Cycle Inhibitor Following the Generation and Screening of an Ascidian-Derived Extract Library Using a Real Time Cell Analyzer
Source: Mar Drugs. 2014 Oct 17;12(10):5222–39. doi: 10.3390/md12105222 (PMC4210896; doi:10.3390/md12105222)
Supplement: Supplementary File 1 [file marinedrugs-12-05222-s001.pdf]

Supplementary Information

Table of Contents

**Table S1**	Cell cycle distribution of MDA-MB-231 cells treated with the active ascidian extracts was determined by flow cytometry through DNA quantification of cells stained with propidium iodide
**Figure S1**	Morphology analysis of MDA-MB-231 cells treated with the indicated ascidian extracts (1 µge/µL) after 24 h seeding
**S1**	Experimental data for the *bis*-TFA salt of eusynstyelamide B (**1**)
**Figure S2**	HPLC UV chromatogram of the active *Didemnum candidum* extract (ascidian code: 114) selected for bioassay-guided fractionation
**Figure S3**	^{1}H NMR spectrum of the *bis*-TFA salt of eusynstyelamide B **(1)** in DMSO-d_6
**Figure S4**	^{13}C NMR spectrum of the *bis*-TFA salt of eusynstyelamide B (**1**) in DMSO-d_6
**Figure S5**	COSY spectrum of the *bis*-TFA salt of eusynstyelamide (**1**) in DMSO-d_6
**Figure S6**	HSQC spectrum of the *bis*-TFA salt of eusynstyelamide (**1**) in DMSO-d_6
**Figure S7**	HMBC spectrum of the *bis*-TFA salt of eusynstyelamide (**1**) in DMSO-d_6
**Figure S8**	Dose response curves for the *bis*-TFA salt of eusynstyelamide B (**1**, EB) in the breast cancer cell line MDA-MB-231 at 72 h post-treatment

Table S1. Cell cycle distribution of MDA-MB-231 cells treated with the active ascidian extracts was determined by flow cytometry through DNA quantification of cells stained with propidium iodide. The results are presented as mean ± SD of triplicates. Significant samples compared to the control are marked with an asterisk * (p < 0.05).

**Extract**	**Sub-G1**		**G0/G1**		**S**		**G2/M**	
	**Mean**	**SD**	**Mean**	**SD**	**Mean**	**SD**	**Mean**	**SD**
DMSO	6.4	1.7	43.1	3.2	52.0	0.9	5.0	2.3
**15**	15.2 *	2.2	18.3 *	1.6	67.6 *	2.3	14.1	3.0
**17**	8.4	0.6	1.4 *	0.2	62.4	0.6	36.2 *	0.3
**29**	5.8	1.5	18.7 *	15.7	69.4 *	11.9	11.9	9.5
**38**	11.1	3.4	19.3 *	2.6	72.2 *	4.1	8.5	2.3
43	5.0	1.7	30.3	4.1	60.5	1.2	9.2	3.0
44	4.4	2.5	33.5	15.6	55.7	6.3	10.8	9.3
53	4.5	0.6	41.1	2.4	52.7	3.6	6.2	1.5
**61**	8.0	1.6	22.4 *	10.4	71.4 *	6.0	6.2	4.4
**63**	17.2 *	6.6	1.3 *	0.8	84.4 *	9.5	14.4	2.0
**71**	3.4	1.0	27.7 *	1.7	66.4 *	0.5	5.9	1.3
**75**	6.4	4.9	4.3 *	2.4	95.7 *	2.4	0.0	0.0
**81**	14.2 *	4.7	3.8 *	1.0	75.1 *	2.0	21.1 *	1.0
**83**	6.8	1.6	16.7 *	6.3	58.5	2.9	24.9 *	9.2
85	6.5	1.9	39.0	0.6	56.0	2.7	5.7	1.4
92	5.3	1.5	31.7	2.8	63.3	4.4	5.0	4.7
**102**	2.1	5.4	28.9 *	0.5	64.9	1.1	6.3	0.6
106	8.6	1.2	51.4	0.8	44.3	1.0	4.3	1.1
**114**	16.4 *	2.7	5.5 *	0.1	69.6 *	1.1	25.0 *	0.8
117	9.7	3.5	54.0	1.6	39.8	2.7	6.1	1.4
128	5.2	1.7	31.6	3.4	58.6	1.2	9.8	2.3
133	7.2	4.2	43.9	7.4	49.8	5.4	6.3	2.1

Figure S1. Morphology analysis of MDA-MB-231 cells treated with the indicated ascidian extracts (200 µge) after 24 h seeding. As controls, cells were treated with complete medium, DMSO (0.1%), or doxorubicin (Dox, 10 µM). The morphology of the cells after treatment was categorized in five groups (A–F). (**A**) Cells not fully attached; (**B**) Cells with typical signs of early stages of cell death; (**C**) Cells well attached to substrate and in contact with other cells; (**D**) Flat and enlarged cells; (**E**) High number of cells detached from substrate. The images were obtained with an OlympusIX70 microscope using a 10× objective. Scale bar = 100 µm.

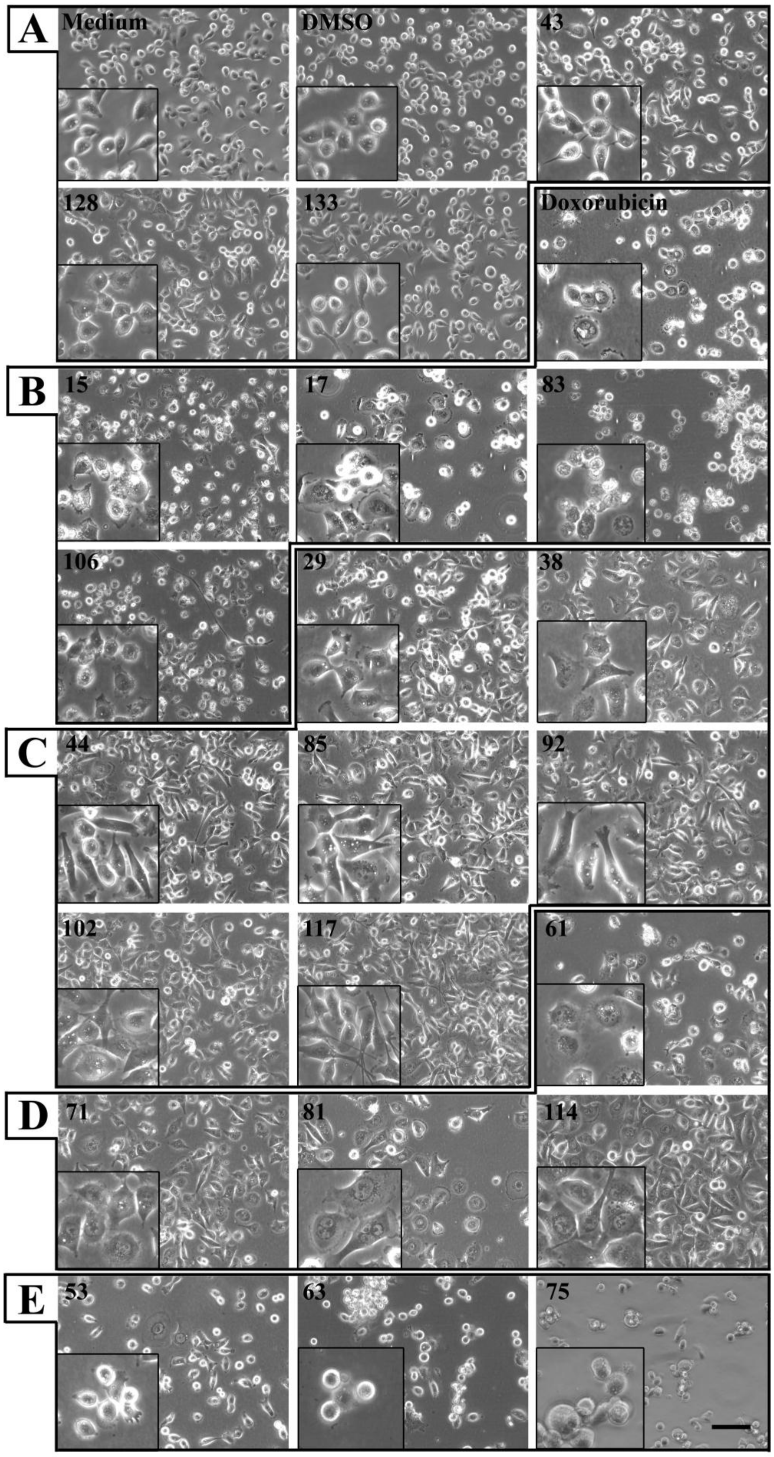

S1. Experimental Data for the *bis*-TFA Salt of Eusynstyelamide B (1)

Bis-TFA salt of eusynstyelamide B (**1**). Isolated as a stable brown gum (3.5 mg); $[\alpha]_D^{26}$ = ±0 (*c* 0.133, MeOH); literature value $[\alpha]_D^{19}$ = ±0 (*c* 0.100, MeOH) [1, 2]; CD (MeOH) λ_{max} (Δε) 208 (−3.0), 227 (−4.6), 298 (−1.1) nm; literature value CD (MeOH) λ_{max} (Δε) 224 (−11) nm [1,2]; ^{1}H NMR [a] (600 MHz, DMSO-d_6) δ_H 10.93 (1H, d, *J* = 2.3, 1-NH), 6.86 (1H, d, *J* = 2.3, H-2), 7.01 (1H, d, *J* = 8.5 Hz, H-4), 6.77 (1H, dd, *J* = 8.5, 1.7 Hz, H-5), 7.45 (1H, d, *J* = 1.7 Hz, H-7), 3.26 (1H, d, *J* = 14.5 Hz, H-8a), 2.78 (1H, d, *J* = 14.5 Hz, H-8b), 6.18 (1H, s, 9-OH), 3.21 (1H, m, H-12a), 2.91 (1H, m, H-12b), 1.48 (2H, m, H-13), 1.40 (2H, tt, *J* = 6.5, 6.5 Hz, H-14), 3.04 (2H, dt, *J* = 5.5, 6.5 Hz, H-15), 7.49 (1H, t, *J* = 5.5 Hz, H-16), 11.15 (1H, d, *J* = 2.4 Hz, 18-NH), 7.65 (1H, d, *J* = 2.4 Hz, H-19), 6.81 (1H, d, *J* = 8.5 Hz, H-21), 6.92 (1H, dd, *J* = 8.5, 1.7 Hz, H-22), 7.51 (1H, d, *J* = 1.7 Hz, H-24), 4.02 (1H, s, H-25), 5.42 (1H, s, 26-OH), 7.72 (1H, t, *J* = 6.0 Hz, H-28), 2.88 (1H, m, H-29a), 2.76 (1H, m, H-29b), 1.12 (2H, m, H-30), 1.12 (2H, m, H-31), 2.87 (2H, m, H-32), 7.37 (1H, t, *J* = 5.8 Hz, H-33); ^{13}C NMR (125 MHz, DMSO-d_6) δ_C 125.6 (C-2), 109.1 (C-3), 126.7 (C-3a), 120.5 (C-4), 120.5 (C-5), 113.6[c] (C-6), 113.3 (C-7), 136.6 (C-7a), 29.0 (C-8), 78.2 (C-9), 175.3 (C-10), 40.2 (C-12), 25.9[d] (C-13), 25.5 (C-14), 40.3 (C-15), 156.7[b] (C-17), 126.7 (C-19), 105.9 (C-20), 127.5 (C-20a), 120.3 (C-21), 120.8 (C-22), 113.5[c] (C-23), 113.5 (C-24), 135.9 (C-24a), 44.0 (C-25), 90.3 (C-26), 168.9 (C-27), 38.4 (C-29), 25.1[d] (C-30), 25.6 (C-31), 40.0 (C-32), 156.6[b] (C-34); (+)-LRESIMS (rel. int.) *m/z* 787 (30%) $[M - 2CF_3COO^- + H]^+$, 789 (100%) $[M - 2CF_3COO^- + H]^+$, 791 (30%) $[M - 2CF_3COO^- + H]^+$. [a] Signals for the exchangeable signals 17-NH_3^+, 17-NH, 34-NH_3^+, and 34-NH were not assigned; [b–d] Signals are interchangeable.

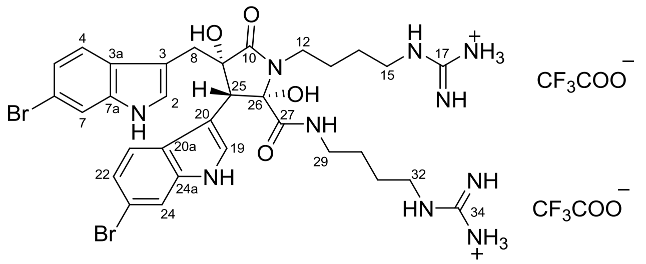

Figure S2. HPLC UV chromatogram of the active *Didemnum candidum* (Extract 114) selected for bioassay-guided fractionation. Every minute of the chromatogram represents one fraction. The yellow shading highlights the active region identified through bioassay-guided fractionation, which corresponds to **1**.

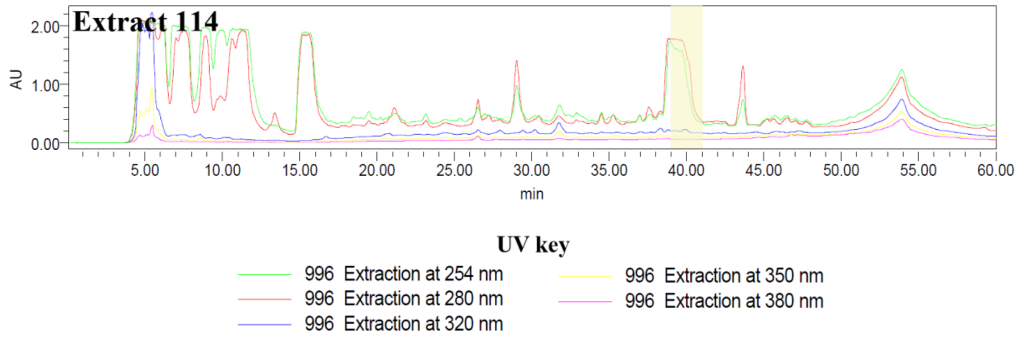

Figure S3. ^{1}H NMR spectrum of the *bis*-TFA salt of eusynstyelamide B (**1**) in DMSO-*d*$_6$.

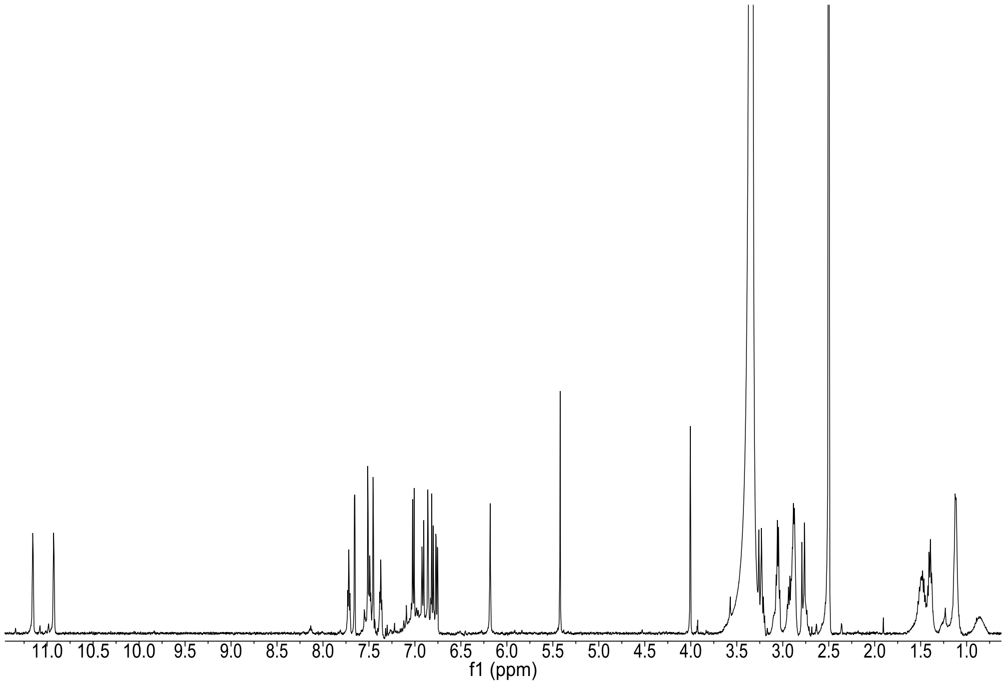

Figure S4. ^{13}C NMR spectrum of the *bis*-TFA salt of eusynstyelamide B (**1**) in DMSO-*d*$_6$.

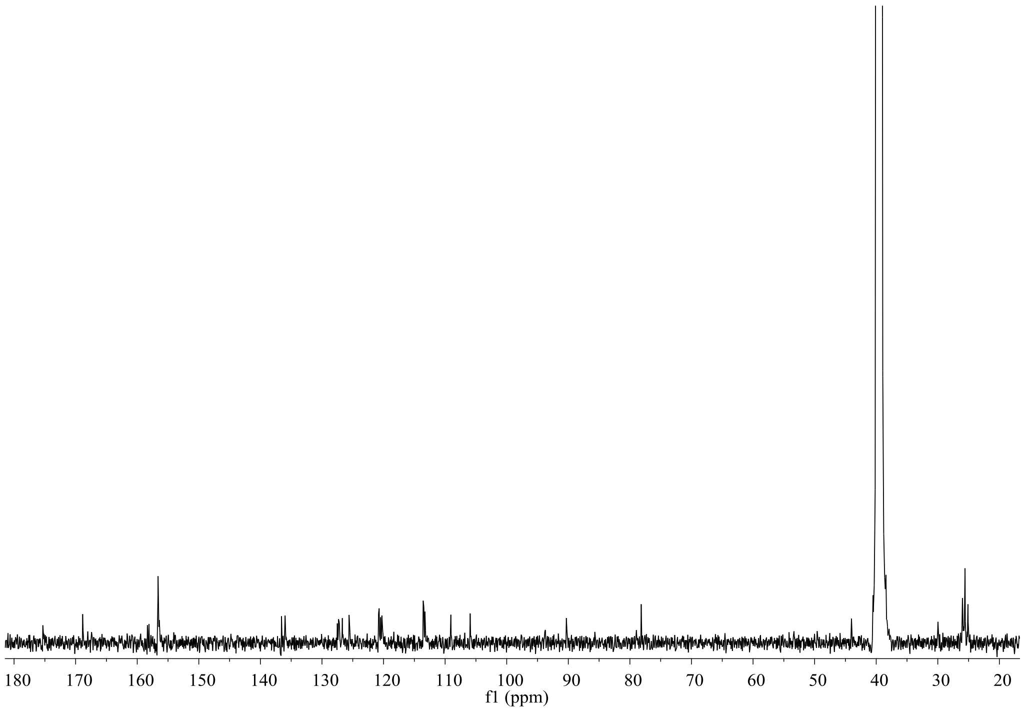

Figure S5. COSY spectrum of the *bis*-TFA salt of eusynstyelamide B (**1**) in DMSO-d_6.

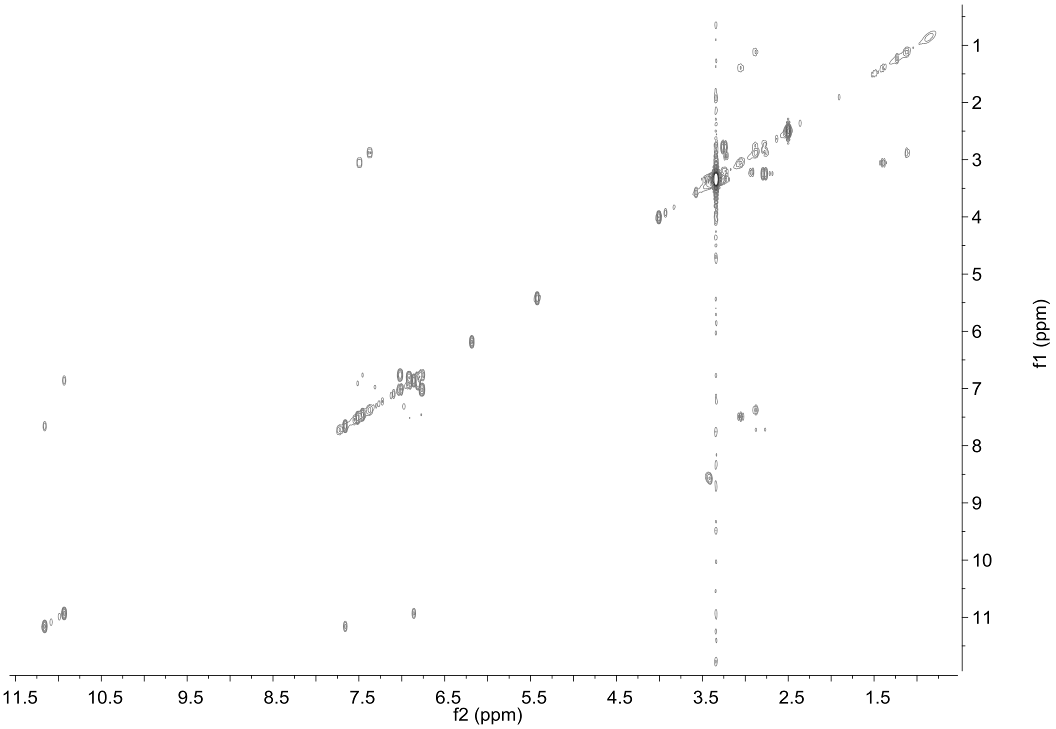

Figure S6. HSQC spectrum of the *bis*-TFA salt of eusynstyelamide B (**1**) in DMSO-d_6.

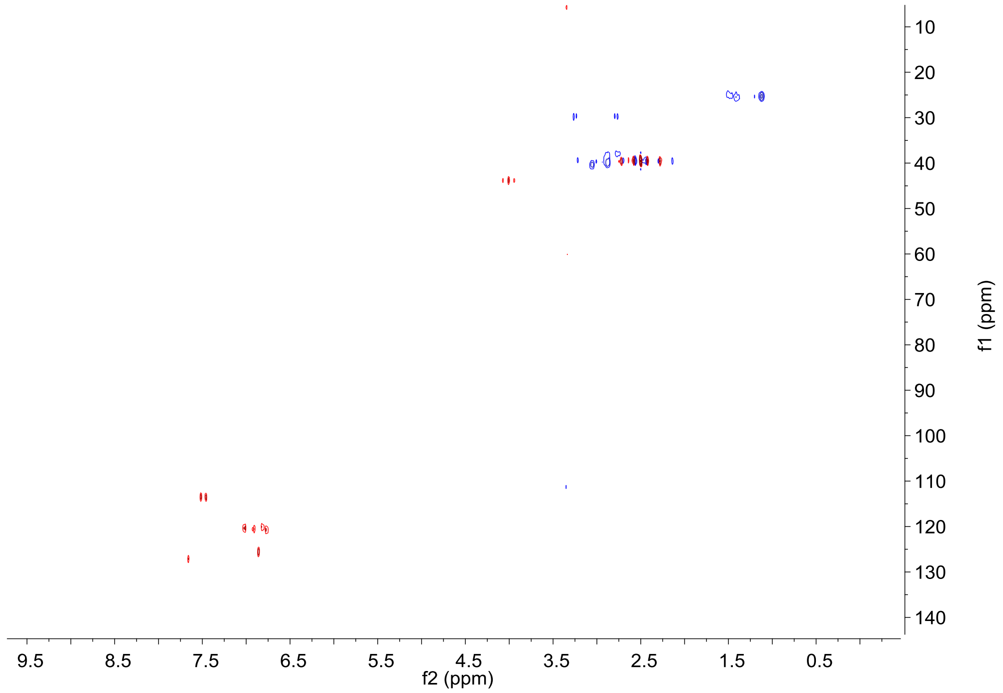

Figure S7. HMBC spectrum of the *bis*-TFA salt of eusynstyelamide B (**1**) in DMSO-d_6.

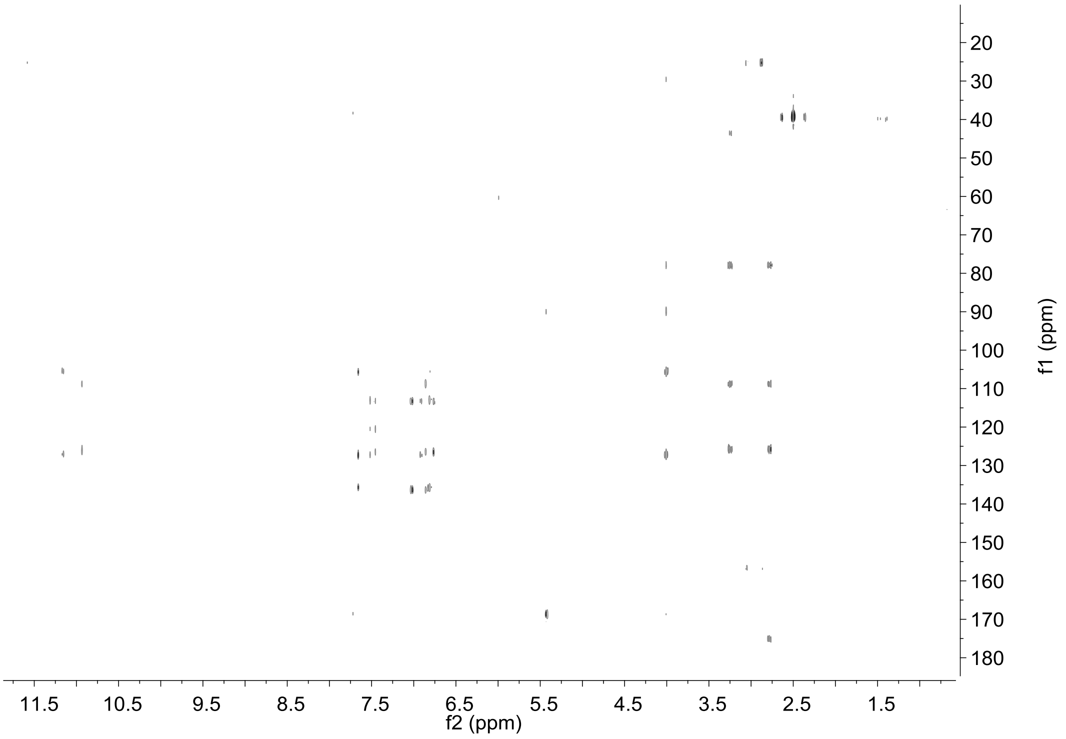

Figure S8. Dose response curves for eusynstyelamide B (**1**, EB) in the breast cancer cell line MDA-MB-231 at 72 h post treatment. The data used to calculate the IC_{50} of **1** in MDA-MB-231 were acquired in an AlamarBlue® assay.

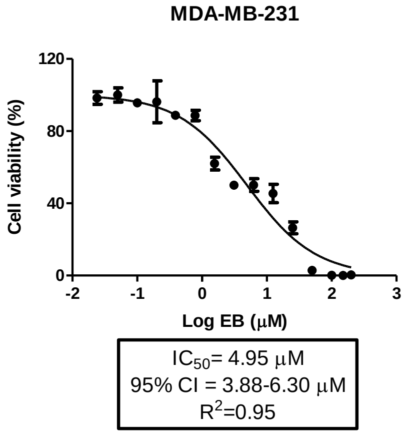

References

1. Tapiolas, D.M.; Bowden, B.F.; Abou-Mansour, E.; Willis, R.H.; Doyle, J.R.; Muirhead, A.N.; Liptrot, C.; Llewellyn, L.E.; Wolff, C.W.W.; Wright, A.D.; *et al*. Eusynstyelamides A, B, and C, nNOS Inhibitors, from the Ascidian *Eusynstyela latericius*. *J. Nat. Prod.* **2009**, *72*, 1115–1120.
2. Tadesse, M.; Tabudravu, J.N.; Jaspars, M.; Strom, M.B.; Hansen, E.; Andersen, J.H.; Kristiansen, P.E.; Haug, T. The antibacterial ent-eusynstyelamide B and eusynstyelamides D, E, and F from the Arctic bryozoan *Tegella cf. spitzbergensis*. *J. Nat. Prod.* **2011**, *74*, 837–841.

© 2014 by the authors; licensee MDPI, Basel, Switzerland. This article is an open access article distributed under the terms and conditions of the Creative Commons Attribution license (http://creativecommons.org/licenses/by/4.0/).
